# Efferocytosis Promotes Suppressive Effects on Dendritic Cells through Prostaglandin E2 Production in the Context of Autoimmunity

**DOI:** 10.1371/journal.pone.0063296

**Published:** 2013-05-15

**Authors:** Irma Pujol-Autonell, Rosa-Maria Ampudia, Raquel Planas, Silvia Marin-Gallen, Jorge Carrascal, Alex Sanchez, Ana Marin, Manuel Puig-Domingo, Ricardo Pujol-Borrell, Joan Verdaguer, Marta Vives-Pi

**Affiliations:** 1 Department of Immunology, Germans Trias i Pujol Research Institute, Autonomous University of Barcelona, Badalona, Spain; 2 Immunology Unit, Dept. of Ciencies Basiques Mediques, University of Lleida & IRBLleida, Lleida, Spain; 3 Research Institute Vall d’Hebron, University Hospital Vall d’Hebron, Barcelona, Spain; 4 Endocrinology and Nutrition Section. University Hospital Germans Trias i Pujol, Badalona, Spain; University of Michigan Medical School, United States of America

## Abstract

**Introduction:**

Efferocytosis is a crucial process by which apoptotic cells are cleared by phagocytes, maintaining immune tolerance to self in the absence of inflammation. Peripheral tolerance, lost in autoimmune processes, may be restored by the administration of autologous dendritic cells loaded with islet apoptotic cells in experimental type 1 diabetes.

**Objective:**

To evaluate tolerogenic properties in dendritic cells induced by the clearance of apoptotic islet cells, thus explaining the re-establishment of tolerance in a context of autoimmunity.

**Methods:**

Bone marrow derived dendritic cells from non-obese diabetic mice, a model of autoimmune diabetes, were generated and pulsed with islet apoptotic cells. The ability of these cells to induce autologous T cell proliferation and to suppress mature dendritic cell function was assessed, together with cytokine production. Microarray experiments were performed using dendritic cells to identify differentially expressed genes after efferocytosis.

**Results:**

Molecular and functional changes in dendritic cells after the capture of apoptotic cells were observed. 1) Impaired ability of dendritic cells to stimulate autologous T cell proliferation after the capture of apoptotic cells even after proinflammatory stimuli, with a cytokine profile typical for immature dendritic cells. 2) Suppressive ability of mature dendritic cell function. 3) Microarray-based gene expression profiling of dendritic cells showed differential expression of genes involved in antigen processing and presentation after efferocytosis. 4) Prostaglandin E2 increased production was responsible for immunosuppressive mechanism of dendritic cells after the capture of apoptotic cells.

**Conclusions:**

The tolerogenic behaviour of dendritic cells after islet cells efferocytosis points to a mechanism of silencing potential autoreactive T cells in the microenvironment of autoimmunity. Our results suggest that dendritic cells may be programmed to induce specific immune tolerance using apoptotic cells; this is a viable strategy for a variety of autoimmune diseases.

## Introduction

The removal of apoptotic cells -i.e. efferocytosis- is crucial in the maintenance of immune tolerance to self. Under physiological conditions, signals from apoptotic cells direct the activation of antigen presenting cells toward a deactivated phenotype [1], [2]. The uptake of apoptotic cells by dendritic cells (DCs), the most potent professional antigen presenting cells for naïve T cells, avoids its maturation resulting in the induction of specific tolerance rather than autoimmunity [3]. However the mechanisms by which efferocytosis induces selective immunosuppression are not fully understood. Deregulated apoptosis or impaired clearance of dying cells favours inflammation and DCs maturation, contributing to chronic inflammation and autoimmune diseases [4], [5].

In a previous work, we demonstrated that immunotherapy with DC loaded with islet apoptotic cells prevented experimental type 1 diabetes (T1D) in non-obese diabetic mice (NOD). Peripheral tolerance to β-cells, lost in autoimmune T1D [6], may be restored through tolerogenic DCs [7]. In fact, this experimental model shows a deficiency in the clearance of apoptotic cells, predisposing them to autoimmunity [8]. When apoptosis of β-cells is experimentally reduced in NOD mice, diabetes is prevented [9], thus indicating that the ratio apoptotic cells/removal is crucial for the maintenance of homeostasis.

Several studies have demonstrated the feasibility of using DCs in clinical immunotherapy [10], [11]. Tolerogenic DCs may be used, as well as blood cells or inert particles, as carriers of autoantigens [12]. This study aims to describe the functional and molecular changes that occur in DCs after islet cell efferocytosis, and demonstrates that tolerogenic DCs acquire suppressive ability which is mediated, at least in part, by an increase in the production of prostaglandin E2 (PGE_2_).

## Materials and Methods

### Ethics Statement

This study was carried out in strict accordance with the recommendations in the Guide for the Care and Use of Laboratory Animals of the Generalitat de Catalunya, Catalan Government. The protocol was approved by the Committee on the Ethics of Animal Experiments of the Germans Trias i Pujol Research Institute (Permit DAAM 5157).

### Mice

Wild-type NOD mice were obtained from The Jackson Laboratory (Bar Harbor, ME, USA) and kept under specific pathogen-free conditions. Only 10- to 14-wk old females were used.

### Cell Lines and Induction of Apoptosis

The NIT-1 cell line, derived from an insulinoma from NOD/Lt mice [13], was chosen because of its expression of β-cell-specific autoantigens (American Type Culture Collection, Manassas, VA). The culture medium used was RPMI-1640 media with 10% FBS (Gibco, Invitrogen, Carlsbad, CA), 100 U/ml penicillin (Normon SA, Madrid, Spain), 100 µg/ml streptomycin (Laboratorio Reig Jofre, Sant Joan Despi, Spain), 2 mmol/l glutamine (Sigma, St. Louis, MO), 1 mmol/l sodium pyruvate (Gibco), and 25 µmol/l β-mercaptoethanol (Sigma). Apoptosis was induced by UV irradiation (10 mJ/m^2^) for 1 hour (FACSCanto II, BD Biosciences, San Jose, CA) and confirmed with annexin V-PE and 7-amino-actinomycin D labelling (7aad) (BD Pharmingen, San Diego, CA).

### Dendritic Cell Generation and Efferocytosis

DCs were generated in vitro from bone marrow progenitors of NOD mice in culture medium containing GM-CSF (1000 U/ml; Prospec, Rehovot, Israel) as previously reported by our group [7]. The DC purity of the culture was evaluated by CD11c-PECy7 staining (BD Pharmingen). The viability was assessed by annexin V and 7aad staining, and cells were counted by flow cytometry (Perfect Count Microspheres, Cytognos, Salamanca, Spain). Efferocytosis was performed by co-culturing DCs with apoptotic NIT-1 pre-labelled with CFSE (Molecular Probes, Invitrogen, Carlsbad, CA) at a 1∶3 ratio for 2 hours. The DCs that captured NIT-1 apoptotic cells (CD11c and CFSE positive cells) -henceforth NITApo-DCs-, were always purified by sorting (FACSAria II, BD Biosciences). Control DCs were either cultured in basal conditions to obtain immature DCs (iDCs) or stimulated with LPS (100 ng/ml; Sigma) for 24 hours to obtain mature DCs (mDCs).

### T Cell Proliferation Assays

NITApo-DCs were co-cultured with isolated splenic T lymphocytes to determine the ability of DCs to induce T cell proliferation after the capture of apoptotic cells. T cells were obtained after mechanical disruption of NOD spleen and purified by negative selection using antibodies to CD19-PE, CD16/32-PE, CD11c-PECy7 (BD Pharmingen), CD11b-PE (ImmunoTools GmbH, Friesoythe, Germany), and Ly-6G(Gr-1)-eFluor660 (eBioscience, CA, USA). The non-stained cell population was purified by sorting (FACSAria II, BD Biosciences). The purity was assessed by CD3-V450, CD8-PerCP-Cy5.5 and CD4-APC-Cy7 (BD Pharmingen) staining, whereas the viability was assessed by annexin V and 7aad staining. iDCs, mDCs or NITApo-DCs (10,000 cells) with 20 µg/ml of insulin (Sigma, St Louis, MO, USA) were co-cultured with 10^5^ T lymphocytes (1∶10 ratio). After 6 days, cells were pulsed with 1 µCi of (^3^H)-thymidine (Perkin Elmer, Waltham, MA, USA) for an additional 16 h. Cells were harvested (Harvester 96, Tomtec Inc., Hamden, CT, USA) and analyzed using a scintillation counter (1450 Microbeta, TriluxWallac, Turku, Finland). T cell proliferation was expressed as c.p.m. In another set of experiments, purified T cells were previously labelled with CFSE and analyzed at day 7 by flow cytometry, as previously described [14]. As a control, T lymphocytes were cultured in basal conditions or with mitogen stimuli (PMA, 25 ng/ml, Sigma) and Ionomycin (IO, 250 ng/ml, Sigma).

### Stability of Tolerogenic Function of DCs after Efferocytosis

To determine the stability of the tolerogenic function of DCs, three maturation stimuli were used in the assays. 5×10^5^ iDCs or NITApo-DCs were cultured for 24 h with LPS (100 ng/ml; Sigma), Poly I:C (0.5 µg/ml; InvivoGen, San Diego, CA, USA), or Zymosan (1 µg/ml; InvivoGen). Cells were washed and counted, and proliferation assays were performed as described above. Results were expressed as T cell proliferation index (T cell proliferation of each condition divided by T cell proliferation induced by iDCs).

### Cytokine Production

The Mouse Th1/Th2/Th17 kit (CBA system; BD Biosciences) was used to assess cytokine production of cytokines by T cells. Culture supernatants from T cell proliferation assays were collected after 7 days and frozen at –80°C until use. IL-2, IL-4, IL-6, IFN-γ, TNF, IL-17A, and IL-10 were measured. Data were analyzed using CBA software. The production of TGF-β production was determined using Human/Mouse TGF-β1 Ready-SET-Go! (eBioscience).

### Assessment of Classical Regulatory T Cells

The amount of CD4^+^, CD25^+^, FoxP3^+^ regulatory T cells (Tregs) was assessed after T cell proliferation experiments. Briefly, 10^5^ purified T cells were co-cultured with autologous iDCs, mDCs or NITApo-DCs at 10∶1 ratio in the presence of insulin. After 7 days, percentages of Tregs were assessed by flow cytometry after membrane staining (CD3-V450, CD8-PerCP-Cy5.5, CD4-APC-Cy7; BD Pharmingen and CD25-PE; eBioscience), permeabilization/fixation (Foxp3 Fixation/Permeabilization Concentrate and Diluent; eBioscience) and intracellular staining (FoxP3-APC; eBioscience).

### Suppression Assays

To assess whether NITApo-DCs have immunosuppressive functions, 10^5^ purified splenic T lymphocytes were co-cultured with 10^4^ autologous mDCs in the presence of iDCs or NITApo-DCs at different ratios (1∶2, 1∶1, 1∶0.5, 1∶0.25) for 7 days with insulin. After 6 days, the cells were pulsed with (^3^H)-thymidine, harvested and counted as described above.

### Transcriptome of Dendritic Cells after Islet Cells Efferocytosis

RNA was obtained from 5×10^5^ NITApo-DCs and sorted iDCs using RNeasy Micro (QIAGEN, Hilden, Germany). Cells from four different mice were used in four paired experiments. Moreover, RNA was also obtained from NIT-1 apoptotic cells. RNA quality (2100 Bioanalyzer, Agilent Technologies Inc., Santa Clara, CA) was optimal for microarray experiments (RNA integrity number >7.6 in all samples). cDNA was synthesized with 50–100 ng of total RNA using the WT expression kit (Ambion, Applied Biosystems, CA, USA), fragmented and labelled with the Terminal labelling kit (Affymetrix, Inc. Santa Clara, CA), purified (GeneChip® Sample Cleanup Module, Affymetrix), and fragmented and checked to verify the integrity. Mouse Gene 1.0 ST Arrays (28.853 genes) were hybridized and scanned by an Affymetrix G3000 GeneArray Scanner.

Raw expression values obtained from CEL files were pre-processed using the Robust Multiarray Averaging method [15]. These normalized values were used for all subsequent analyses. Experimental data have been uploaded into ArrayExpress for the European Bioinformatics Institute (EBI, www.ebi.ac.uk/aerep/login; Username: Reviewer_E-MEXP-3374 and accession number: xC6224PP). Data were subjected to non-specific filtering to remove low signal and low variability genes. Conservative (low) thresholds were used to reduce possible false negative results. The selection of differentially expressed genes was based on a linear model analysis with empirical Bayes modification for the variance estimates, as described [16]. This method is similar to using a ‘t-test’ with an improved estimate of the variance. To account for the multiple testing probability effects arising when many tests (one per gene) are performed simultaneously, p-values were adjusted to obtain a strong control over the false discovery rate using the Benjamini-Hochberg method [17]. Genes with a p-value ≤0.002, adjusted p-value ≤0.08 and fold change (FC) ≥1.38 were considered upregulated, whereas genes with FC ≤ −1.37 were considered downregulated. The Ingenuity Pathway Analysis (IPA) (Ingenuity Systems ®) was used to identify the canonical pathways from the IPA library that were most significant to the data sets.

### Quantitative RT-PCR

Total RNA from each sample (<100 ng) was reverse transcribed with a High Capacity cDNA Reverse Transcription Kit (Applied Biosystems). cDNA was preamplified with the TaqMan PreAmp Master Mix Kit (Applied Biosystems), for each gene-specific target using a pool of TaqMan Gene Expression Assays as a source of primers. This preamplification reaction generated approximately a 1.000- to 16.000-fold increase in each gene-specific target without inducing any bias. The resulting preamplified material was diluted and used as the starting material for the subsequent singleplex RT-PCR, performed on a LightCycler® 480 (Roche, Mannheim, Germany). Quantitative PCR (qPCR) assays were performed under TaqMan universal assay conditions and using the following TaqMan Assays: *Ccr7* (Mm01301785_m1), *Ccl5* (Mm01302428_m1), *Cd74* (Mm00658576_m1), *Cd83* (Mm00486868_m1), *Il2ra* (Mm01340213_m1), *Tnfrsf9* (Mm00441899_m1), *Ins2* (Mm00731595_gH) and *Iapp* (Mm00439403_m1). The relative quantification was determined by normalizing the expression for each gene of interest to the housekeeping gene *Gapdh* (Mm99999915_g1), as described in the 2^−ΔCt^ method [18].

### Prostaglandin E2 Quantification

Based on microarray results, we assessed the production of Prostaglandin E2 (PGE_2_) by NITApo-DCs, iDCs and NIT-1 apoptotic cells as controls. With this purpose, supernatants of different cultures were collected after 24 hours of culture and frozen at –80°C until use. The assessment of PGE_2_ was performed by ELISA (PGE_2_ EIA Kit-Monoclonal; Cayman Chemicals, Ann Arbor, MI). Limit of detection: 80% B/B_0_∶15 pg/ml. Sensitivity: 50% B/B_0_∶50 pg/ml. Results were expressed as an index (pg PGE_2_/10^6^ cells). In order to validate the results, gene expression level was assessed by qRT-PCR using the following TaqMan Assays: *Ptgs1* (Mm00477214_m1), *Ptgs2* (Mm00478374-m1), *Alox15* (Mm00507789_m1) and *Ltc4s* (Mm00521864_m1) as described above.

### Role of PGE_2_ in Suppression Assays

To confirm the role of PGE_2_ in suppressive function of NITApo-DCs (see above, Suppression Assays), T cell proliferation experiments were performed using a specific-COX2 inhibitor (NS-398, Sigma) that inhibits PGE_2_ production. NS-398 was added to co- suppression assays at 10 µM. T cell proliferation was measured using (^3^H)-thymidine and expressed as c.p.m. as described above. Moreover, to determine if the mechanism depends on cell-cell contact, supernatants from NITApo-DCs cultures, in which PGE_2_ concentration was previously measured by ELISA (PGE_2_ EIA Kit-Monoclonal; Cayman Chemicals), were added to suppression assays instead of NITApo cells in a final concentration of 50 and 250 pg/ml.

### Statistical Analysis

Statistics was performed using the Prism 5.0 software (GraphPad software Inc., San Diego, CA). For paired data, a non-parametric Wilcoxon test was performed. Otherwise, Mann Whitney test was used. A p-value <0.05 was considered significant.

## Results

### Impairment of DCs to Stimulate Autologous T cell Proliferation after the Capture of Apoptotic Cells Even after Proinflammatory Stimuli

**Figure 1:**
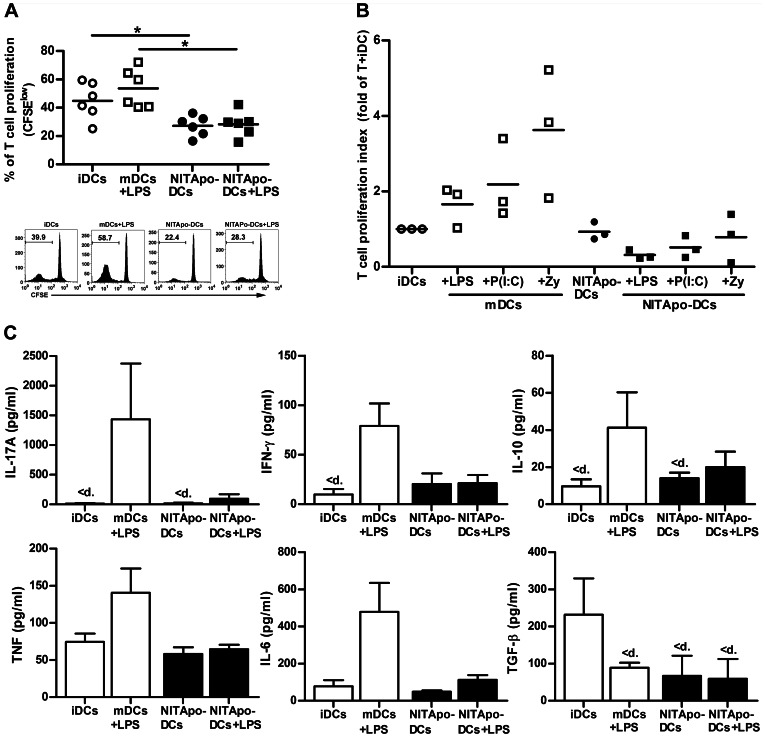
Impaired ability of DCs to stimulate autologous T cell proliferation after the capture of apoptotic cells, even after proinflammatory stimuli and changes cytokine profile. **A)** Top: Autologous proliferation of T cells (% proliferation using CFSE) after stimulation induced by immature DCs (iDCs, white circles) and by DCs loaded with NIT-1 apoptotic cells (NITApo-DCs, black circles) with insulin (20 µg/ml) at a ratio of 1∶10 for 7 days. iDCs and NITApo-DCs were previously activated with inflammatory stimulus LPS (100 ng/ml, squares) during 24 h. Lines show the mean of six independent experiments for each condition. Bottom: A representative flow cytometry histogram is shown from six experiments performed. **B)** Autologous T cell proliferation (c.p.m. for ^3^H thymidine assay) induced by iDCs and mDCs (white circles and squares) and NITApo-DCs (black circles and squares) with insulin (20 µg/ml) at a ratio of 1∶10 for 7 days. iDCs and NITApo-DCs were previously activated with proinflammatory stimuli LPS (100 ng/ml), Poly I:C (P(I:C), 0.5 µg/ml) and Zymosan (Zy, 1 µg/ml) during 24 h. Lines show the mean of three independent experiments represented as T cell proliferation index (T cell proliferation of each condition divided by T cell proliferation induced by iDCs, mean). **C)** Cytokine production in T cell proliferation experiments. Levels of IL-17A, IL-10, IFN-γ, TNF and IL-6 were measured in supernatants from autologous T cell proliferation experiments induced by iDCs and mDCs (white bars) or by NITApo-DCs (black bars) in basal conditions or in the presence of LPS for 24 hours. Results are expressed as mean+SEM from four independent. Double-sided Wilcoxon test was used for the evaluation of statistical significance (* *P*<0.05). <d means values below the standard.

A key feature of tolerogenic DCs is their low capacity for priming T cells. Autologous T cell proliferation assays were performed to assess the ability of efferocytosis to generate tolerogenic DCs. DCs generated from bone marrow progenitors were >80% pure, based on staining for the DC marker CD11c, and viability was always >90%. After purification by sorting, NITApo-DCs were >99% pure, and viability was >86%. T cell purity and viability were always over 90% and 95% respectively (data not shown). We observed that the capture of apoptotic cells by DCs significantly impairs T cell proliferation when compared with immature DCs (Figure 1A). After LPS stimulus, NITApo-DCs induce a T cell proliferation percentage similar to that of non-stimulated NITApo-DCs (Figure 1A), and statistically different to T cell proliferation induced by mDCs previously activated with LPS. We used (^3^H)-thymidine to validate these results and to determine the effect of two additional proinflammatory stimuli -Poly I:C and Zymosan- to simulate both viral infection and inflammation, respectively (Figure 1B). The results indicate that the proliferation of T cells induced by NITApo-DCs does not increase, not even after the effect of these proinflammatory stimuli.

### Cytokine Profile after Efferocytosis is Similar to iDCs, and Stable after LPS Stimulus

Cytometric Bead Array (CBA) analysis showed that NITApo-DCs display a cytokine profile (IFN-γ, IL-17A, IL-10, IL-6 and TNF) similar to iDCs (Figure 1C). In contrast, the production of TGFβ was higher in iDCs than in NITApo-DCs, in which TGFβ concentration was below the limit of detection. IL-2 and IL-4 were not detected in any condition of the assay. This cytokine profile induced by NITApo-DCs was stable after proinflammatory stimulus (LPS). T cells co-cultured with DCs matured with LPS, displayed a biological, although non significant, increase of IL-17, IFN-γ, TNF and IL-6. As expected, the production of TGFβ was inhibited after LPS stimulation in mDCs.

### CD4+ CD25+ FoxP3+ Classical Regulatory T Cells Subset is not Increased after DC Efferocytosis

**Figure 2:**
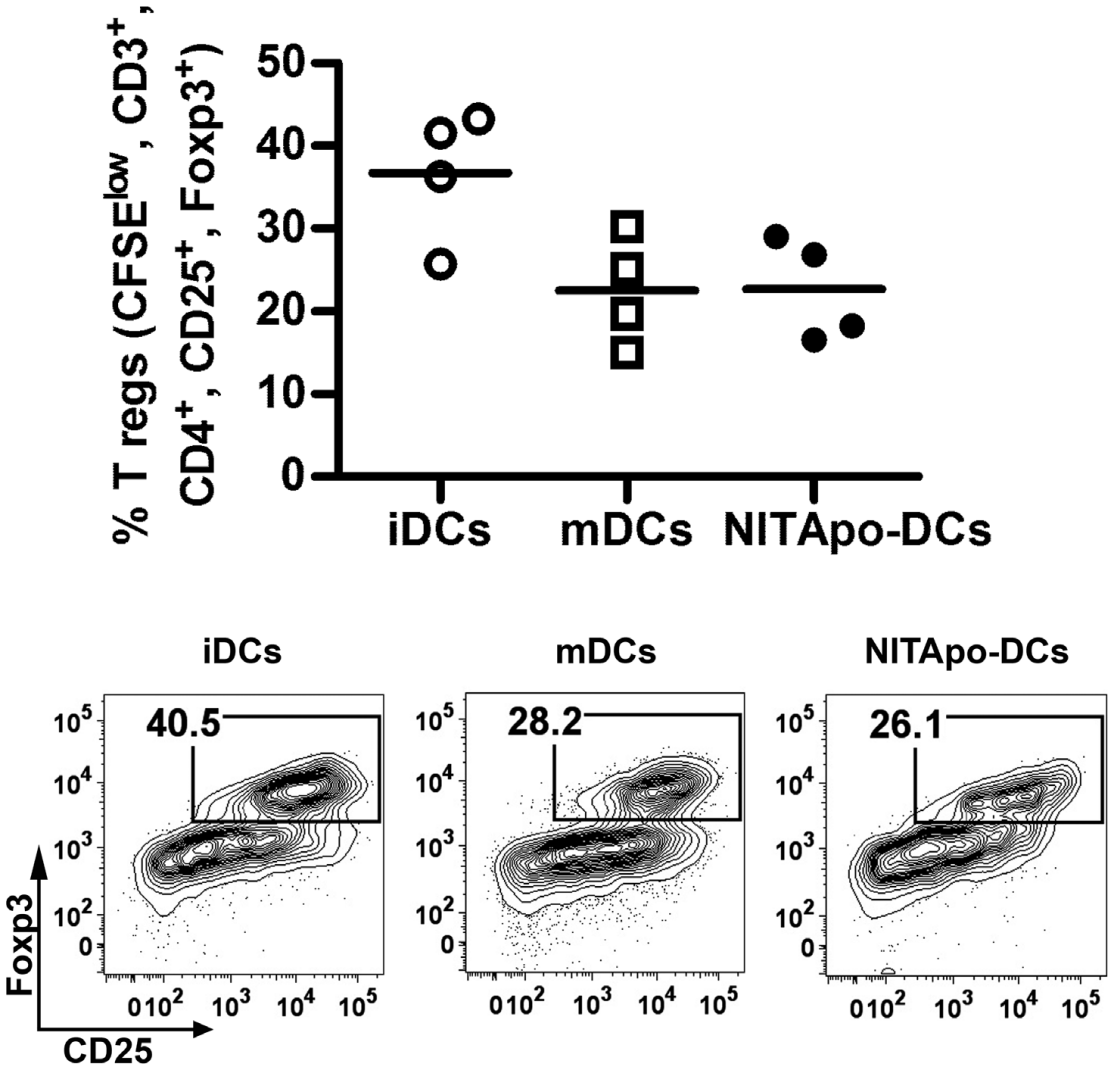
CD4+ CD25+ FoxP3+ classical regulatory T cells subset in not increased by DCs after efferocytosis. Top: Percentage of proliferating CFSE^low^, CD3^+^, CD4^+^,CD25^+^, FoxP3^+^ regulatory T cells in autologous T cell proliferation assays with immature DCs (iDCs, white circles), mature DCs (mDCs, white squares) and DCs loaded with apoptotic cells (NITApo-DCs, black circles) with insulin (20 µg/ml) at a ratio of 1∶10 for 7 days. Plots show the mean (line) of four independent experiments. Double-sided Wilcoxon test was used for the evaluation of statistical significance. Bottom: Representative FACS plots showing CD25^+^ FoxP3^+^ regulatory T cells gated on CFSE^low^, CD3^+^, CD4^+^.

In vitro proliferation assays demonstrated that T cell hyporesponsiveness induced by NITApo-DCs was not due to an increase in CD4+ CD25+ FoxP3+ regulatory T cells. As shown in Figure 2, the percentage of proliferating classical regulatory T cells induced by NITApo-DCs was not higher than those induced by iDCs or mDCs.

### Efferocytosis Promotes Suppressive Effects in Dendritic Cells

**Figure 3:**
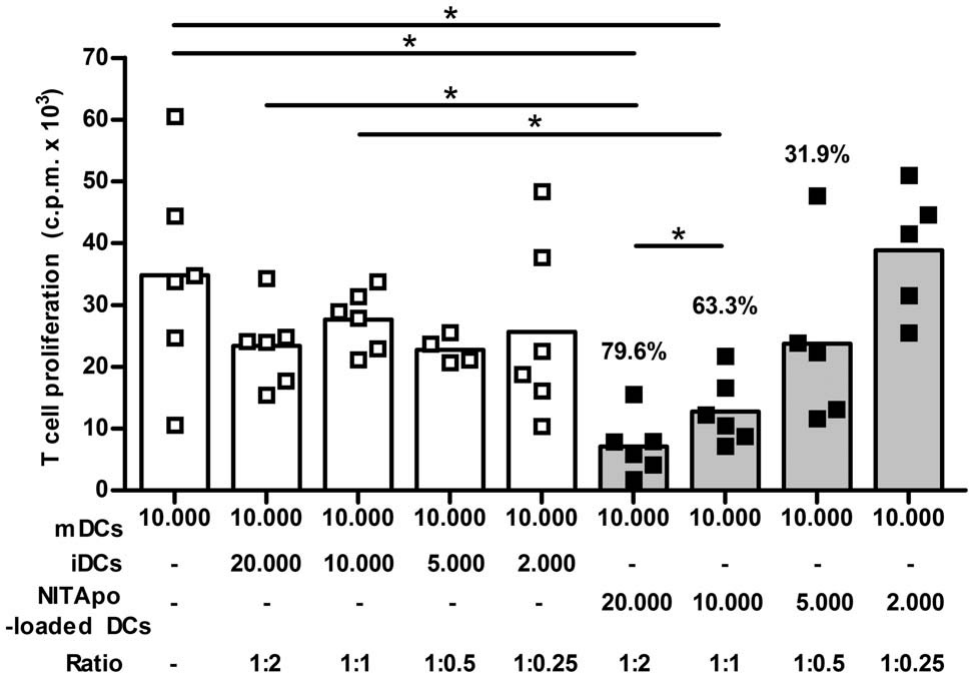
Suppressive effects of NITApo-DCs on mDCs ability to induce T cell proliferation. Autologous T cell proliferation (c.p.m. for ^3^H thymidine assay) induced by mature DCs (mDCs, white bar) in the presence of immature DC (iDCs, white bars) or DCs loaded with NIT-1 apoptotic cells (NITApo-DC, grey bars) with insulin (20 µg/ml) at different ratios (from 1∶2 to 1∶0.25) for 7 days. Percentage of inhibition is given on top of histogram bars. Results from six independent experiments. Bars and squares represent means and individual experiments respectively. Double-sided Wilcoxon test was used for the evaluation of statistical significance (* *P*<0.05).

The suppressive effects of NITApo-DC on the capacity of DCs to induce T cell proliferation after efferocytosis were determined. The percentage of proliferating T cells stimulated with mDCs was not affected by the presence of iDCs at different ratios (from 1∶2 to 1∶0.25). In contrast, when NITApo-DCs were added to T cells cultured with mDC, we observed a reduction in T cell proliferation (Figure 3) in a dose-dependent manner up to 79.6% reduction at a ratio of 1∶2, when compared to T cell proliferation induced by mDCs (p<0.05). In this condition, the effect depended on the dose of NITApo-DCs, showing significant differences at 1∶2 and 1∶1 ratios.

### Gene Expression Profile of DCs after Efferocytosis

Microarray analysis was performed with sorted NITApo-DCs and iDCs. Bioinformatic analysis was performed, taking into account genes that showed a p value <0.002, adjusted p value <0.083. 278 genes out of the 28,853 mouse genes represented in the gene chip were differentially expressed in NITApo-DCs when compared to iDCs. In addition, 177 (64%) out of these 278 genes were downregulated, and the remaining 101 (36%) were upregulated.

We analyzed several categories and molecules related to immune tolerance to diabetogenic autoantigens (Table 1). Differentially expressed genes from the immune system were mainly downregulated and involved in antigen processing, presentation and coestimulation (Table S1). We found a downregulation in chemokine and chemokine receptor gene expression involved in T cell recruitment. In contrast, chemokine genes involved in phagocytosis and phagocyte recruitment were upregulated. Other categories with genes with altered expression were natural immunity, immunoregulation, transcription factors and signalling; this fits well with an inhibition of DC maturation and T cell coestimulation/activation. In addition to these changes in the expression of genes with immune response function, genes in the metabolism category showed expression changes after apoptotic cell uptake (Table S1). The eicosanoids biosynthesis from the arachidonic acid pathway was altered: two genes involved in the PGE_2_ synthesis pathway (*Ptgs2* and *Ptges*) were upregulated, and two other genes involved in leukotriene synthesis from arachidonic acid (*Alox14* and *Ltc4s*) were downmodulated. Specific transcripts for islet cells were found increased in DCs after the engulfment of apoptotic cells, some of them being T1D autoantigens (*Cpe, Iapp, Ins1, Ins2, Sst, Tspan7*). Validation by qRT-PCR of the eight selected targets confirmed the microarray findings (Figure S1). As expected, *Ins2* and *Iapp* expression were not found in iDCs before efferocytosis of apoptotic islet cells.

**Table 1:** Differentially expressed genes related to immune tolerance to diabetogenic autoantigens.

Categories	P val	Genes
Adhesion	<0.001330	Adora3, Cd34, Cd69, Cldn1, Pdpn
Antigen Presentation	<0.000757	Cd74, Il4i1, H2-Ab1, H2-DMb2, H2-Eb1, H2-M2, Rab27a
Chemokines	<0.001271	Ccl12, Ccl17, Ccl2, Ccl22, Ccl3, Ccl4, Ccl5, Ccl7, Ccr2, Ccr7, Cx3cl1, Cxcl1, Cxcl5, Cxcr2, Ppbp
Coestimulation	<0.001957	Cd80, Cd83, Cd86
Cytokines	<0.000001	Il1a, Il2ra, Tnf, Tnfsf4
Immunoregulation	<0.000215	Ly9, Serpinb2, Serpinb8, Slamf6, Slamf7
Islet cells	<0.000190	Cpe, Iapp, Ins1, Ins2, Sst, Tspan7
Metabolism	<0.001269	Alox15, Cacnb3, Fscn1, Ltc4s, Ptges, Ptgs2
Natural Immunity	<0.001838	Cd209a, Cd300e, Marco, Mrc1
Signaling	<0.000975	Jak2, Mapk13, Pik3cg, Samsn1
Transcription Factor	<0.000022	Irf4, Nr4a3, Stat4, Xbp1

### Suppressive Effects of Dendritic Cells after Efferocytosis Involve Prostaglandin E2 Production

**Figure 4:**
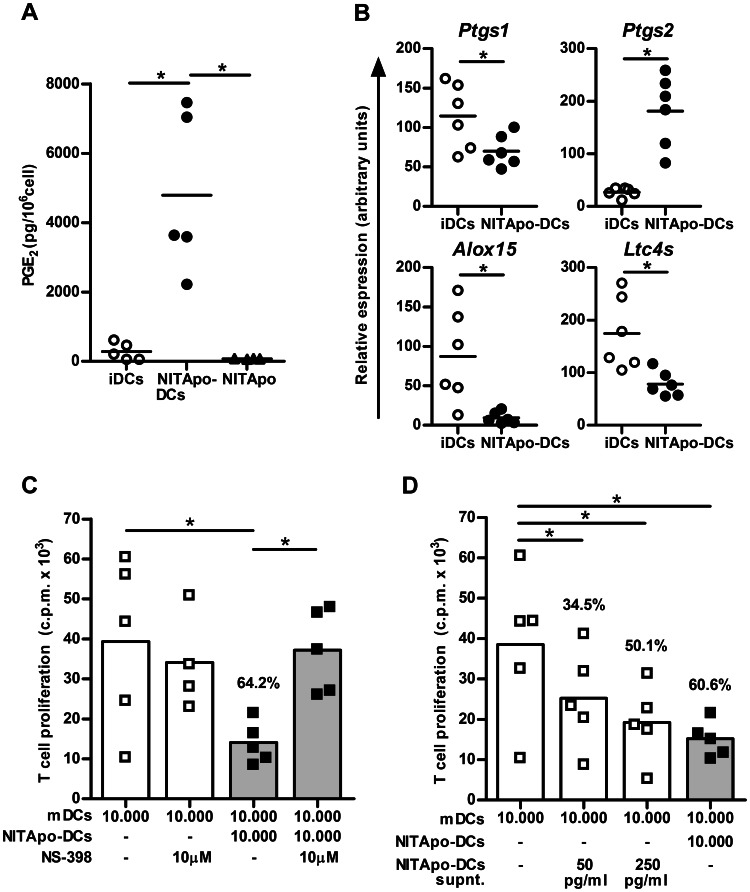
Effect of prostaglandin E2 produced by NITApo-DCs on T cell proliferation. **A)** Quantification of the PGE_2_ by ELISA in culture supernatants of immature DCs (iDCs, white circles), DCs loaded with apoptotic cells (NITApo-DCs, black circles) and apoptotic NIT-1 cells (NITApo, black triangles). ELISA data are represented as pg/10^6^ cells. Plots show the mean (line) of five independent experiments. **B)** Quantitative RT-PCR results for *Ptgs1, Ptgs2, Alox15* and *Ltc4s* genes in iDCs (white circles) and in NITApo-DCs (black circles). Gene expression signals were normalized to *gapdh*. Plots show the mean (line) of six independent experiments. **C)** Autologous T cell proliferation (c.p.m. for ^3^H thymidine assay) induced by mDCs (white bars) in the presence of NITApo-DC (grey bars) with insulin (20 µg/ml) at a ratio of 1∶1 for 7 days. NS-398, specific-COX2 inhibitor (10 µM) was added to block PGE_2_ production and reverse the suppressive effect of NITApo-DCs. Percentage of inhibition is given on top of histogram bar. Results from five independent experiments. **D)** Autologous T cell proliferation (c.p.m. for ^3^H thymidine assay) induced by mDCs (white bar) in the presence of NITApo-DC (grey bar) with insulin (20 µg/ml) at a ratio of 1∶1 for 7 days. To determine if the mechanism depends on cell-cell contact, supernatants from NITApo-DCs cultures, (white bars, 50 and 250 pg/ml of PGE_2_) were added to suppression assays instead of NITApo-DCs. Percentage of inhibition is given on top of histogram bars. Results from five independent experiments (Mean). Bars and symbols represent mean and individual experiments, respectively. One-sided Wilcoxon test was used for the evaluation of statistical significance. Symbol * marks statistically significant differences, p<0.05.

Based on microarray results we examined the production of PGE_2_ by NITApo-DCs by ELISA. The concentration of PGE_2_ was significantly increased in the supernatant of NITApo-DCs cultures when compared to iDCs (p<0.05) (Figure 4A). However, the concentration of PGE_2_ in the supernatant of NIT-1 cells after the induction of apoptosis was very low, thus ruling out that the increase in PGE_2_ production comes from these cells. These results agree with microarray data and were validated by qRT-PCR (Figure 4B). The expression of *Ptgs2* gene (encoding for COX-2) was significantly higher (p<0.05) in DCs after efferocytosis. Furthermore, the expression of *Ptgs1, Alox15* and *Ltc4s* genes -encoding for COX-1, Arachidonate 15-lipoxygenase and Leukotriene C4 synthase respectively- was significantly lower in DCs after the capture of apoptotic cells (p<0.05).

To demonstrate the role of PGE_2_ in the suppressive function of NITApo-DCs (see above), T cell proliferation experiments were performed using a specific-COX-2 inhibitor (NS-398) that inhibits PGE_2_ production. First, we confirmed that NS-398 inhibits the production of PGE_2_ (data not shown). By inhibiting the activity of COX-2, the effect of efferocytosis on DCs in T cell proliferation was reverted (p<0.05) (Figure 4C), reaching similar levels of T cell proliferation induced by mDCs. NS-398 had no effect on T cell proliferation induced by mDCs. These results indicate that PGE_2_ is involved in the suppression ability of NITApo-DCs. Finally, we tested the effect of the supernatant of NITApo-DCs cultures in the suppression of T cell proliferation induced by mDCs. We found that the addition of supernatant from NITApo-DCs (50 and 250 pg/ml of PGE_2_) to T cell proliferation cultures significantly decreases T cell proliferation (Figure 4D), reaching levels of suppression similar to those induced by NITApo-DCs with similar PGE_2_ concentration (34.5%, 50.1% and 60.6% respectively). Moreover, a high concentration of pure PGE_2_ added to the cultures (50 ng/ml) resulted in an inhibition >90.9% (data not shown). These data confirm the role of PGE_2_ released by DCs in the suppressive effects of efferocytosis in a cell-cell contact independent way.

## Discussion

Apoptotic cells serve a dual purpose: they prevent the spreading of cellular debris into the extracellular milieu, and when engulfed by antigen presenting cells, such as DCs, they contribute to the maintenance of self-tolerance through the presentation of autoantigens in an active ‘suppressive’ process that constitutes a silencer event [19]. DCs can be modified ex-vivo, targeted with different antigen delivery systems and used as ‘vaccines’ to induce or restore tolerance [20]. A greater understanding of the tolerogenic mechanisms is important for therapeutic purposes to prevent autoimmune processes. In this study we provide evidence that immature DCs from NOD mice -a spontaneous model of autoimmune diabetes- engulf apoptotic islet cells thus resulting in tolerogenic functions and immunosuppressive ability through PGE_2_ production.

The strongest evidence linking efferocytosis with tolerance to self is the association of autoimmune diseases and defective apoptotic cell clearance [21], [22]. We previously demonstrated that peripheral tolerance may be restored by the administration of dendritic cells loaded with islet apoptotic cells in experimental autoimmune diabetes [7]. T1D prevention in NOD mice was achieved only when DCs were loaded with apoptotic islet cells but not with fibroblasts, hence confirming the antigen specificity of immunotherapy. In general, tolerance re-establishment could be induced by the deletion or anergy of autoreactive T lymphocytes after class I and class II presentation [23], [24], by the expansion of B and T regulatory cells [25], [26] or by alternative mechanisms. DCs have a crucial role in diabetes in NOD mice, a well-established model of T1D that shares multiple characteristics with human disease [27]. However, we have to keep in mind the limitations of the NOD mouse model and the intrinsic defects in dendritic cells [28].

During normal cell turnover, apoptotic cells are removed by phagocytes, but also by other cell types including epithelial cells [29]. In the absence of inflammation, antigen presenting cells will not receive maturation stimuli, and after islet cell efferocytosis, they should enter the afferent lymph node and inhibit autoreactive T cell activation. This has important implications for the design of therapies to re-establish tolerance using DCs as carriers of autoantigens. In fact, in NOD mice, the DCs injected intraperitoneally migrate to peritoneal lymph nodes, particularly in the pancreatic lymph nodes, and the cell signal remains up to one week [30].

These DCs that *in vivo* induce tolerance, were *in vitro* characterized in T cell proliferation assays. The whole T cell response was analyzed because DCs present exogenous antigens to CD4+ T cell subset and efficiently cross-present exogenous antigens to CD8+ T cells. The results confirm the impairment of autologous T cell response after efferocytosis. We are well aware of the low T cell proliferation index in basal conditions, probably due to experimental design which uses NOD mice, and the whole T cell repertoire instead of islet specific systems, as described [31]. Moreover, the tolerogenic function acquired by DCs after efferocytosis is stable and resistant to inflammation; this is a very important feature to take into account for future therapy design. In terms of cytokine secretion, a low IL-6, TNF-α, IFN-γ and IL-17 phenotype partially mediates their effect in re-establishing peripheral tolerance. IL-12 production was not determined because a defect in the secretion of this cytokine has been described in bone marrow DCs of NOD mice [32]. It has been reported that the blockade of IL-17 prevented diabetes in NOD mice [33]. Nevertheless, the absence of TGF-β secretion, together with low IL-10 production, both cytokines related to immune suppression, suggests that the mechanism of immunological tolerance mediated by classical regulatory T cells is not involved in the process. In fact, an increase of CD4^+^ CD25^+^ FoxP3^+^ regulatory T cells was not observed thus suggesting that the induction of tolerance is not dependent on the increase in this T cell subset, as described in other tolerance assays [34].

Interestingly, we found that efferocytosis promotes the suppression of mature DC function, a described mechanism of tolerance [35]. Our results demonstrated that the effect was dose dependent thus indicating that there is an active mechanism of suppression by tolerogenic DCs. Microarray data support this mechanism and show overexpression of two genes related to the pathway of PGE_2_ synthesis (*Ptgs2* and *Ptges*). PGE_2_ depresses cellular immunity [36], inhibits T-cell proliferation [37], induces T regulatory cell function [38], and contributes to insulitis suppression in NOD mice [39]. It is well known that the removal of apoptotic cells by macrophages is crucial for the active suppression of inflammation, through several mechanisms that involve PGE_2_ [40]. We observed that PGE_2_ produced after efferocytosis decrease T cell proliferation induced by mature DCs. This suppressive effect is dose dependent and cell-cell contact independent. The blockade of PGE_2_ production has no effect on T cell proliferation induced by mDCs but avoids the suppressive function of DCs after efferocytosis, thus confirming that PGE_2_ is responsible for this effect. Furthermore, the genes involved in the synthesis of leukotriene from arachidonic acid, such as *Alox15* and *Ltc4s*, were downmodulated after efferocytosis. Leukotrienes are mediators of inflammation and their excessive production has been associated with inflammatory and autoimmune disorders [41]. Since prostaglandins activate the peroxisome proliferator-activated receptor **(**PPAR) γ-dependent pathway [42], these molecules may contribute to the tolerogenic potential of these cells. Ingenuity analysis of microarray data showed that the canonical pathway of the PPAR is altered in DCs after efferocytosis. This transcription factor is responsive to the lipid status of the cell and has a determinant role in the engulfment of apoptotic cells, negatively regulating DC maturation and avoiding the autoimmune attack against dying cells [43].

Gene expression profiles of tolerogenic DCs have been previously reported [44], [45], [46]. However, the transcriptome of DCs after efferocytosis was still unknown. In this study we revealed new altered suppression pathways that confirm DCs tolerogenic phenotype. The decrease in the expression of antigen processing, presentation and coestimulation related genes was confirmed. The immune response gene profile indicates that DCs remain immature, but not inactive, after efferocytosis. Endocytosis-related genes were found upregulated, a feature of tolerogenic DCs [47]. The downregulation of CCL5, imprint of DC maturation, can be a regulatory effect of PGE2 as previously described [48]. This fact, toghether with the decreased expression of CCL17 and CCL22 genes both related to T cell recruitment, may alter the lymphocyte subsets recruited by tolerogenic DCs. The expression of chemokine receptor genes related to DCs migratory function was downregulated, in apparent contrast to the previously described upregulation of CCR7 after efferocytosis [3]. Our results can be explained by the reported dissociation between CCR7 membrane expression and mRNA amounts [49].

It is well known that DCs pulsed with apoptotic cells can induce anti-viral and anti-tumor antigen specific immunity [50]. This is in apparent contradiction to our results and could be explained by differences on the antigenic content of apoptotic cells and their connection with autoimmunity. The avidity of anti-tumoral T lymphocytes should be higher than that of autoreactive T cells that escape from central tolerance [51].

In conclusion, the tolerogenic behaviour of DCs after the uptake of apoptotic cells suggests a mechanism of silencing potential autoreactive T cells in the microenvironment of autoimmunity. This mechanism is mediated, at least in part, through PGE_2_ production. In this context, efferocytosis is an active silencer event in DCs and not a passive lack of maturation and cytokine production. In summary, DCs seem to recognize apoptotic cells as a source of autoantigens and induce regulatory mechanisms in the islet milieu to maintain peripheral tolerance to self. The importance of this physiological mechanism in the prevention of autoimmunity may play a critical role as a booster shot in specific immune tolerance.

## Supporting Information

Figure S1 **Quantitative RT-PCR validates the microarray results.** Histograms represent quantitative RT-PCR results for the selected genes in DCs (iDCs, white bars) and in DCs after the engulfment of NIT-1 apoptotic bodies (NITApo-DCs, black bars). Gene expression signals were normalized to GAPDH. Results from eight independent experiments. One-sided Wilcoxon’s test was used for the evaluation of statistical significance. Symbols * and ** mark statistically significant differences, p<0.05 and p<0.01. Data with values below limit of detection are marked with <d.
(TIF)

Table S1 **Differentially expressed genes in DCs after efferocytosis of islet cells.**
(DOCX)

## References

[1] Erwig LP, Henson PM (2007) Immunological consequences of apoptotic cell phagocytosis. Am J Pathol 171: 2–8. 17591947 10.2353/ajpath.2007.070135 PMC1941587

[2] Green DR, Ferguson T, Zitvogel L, Kroemer G (2009) Immunogenic and tolerogenic cell death. Nat Rev Immunol 9: 353–363. 19365408 10.1038/nri2545 PMC2818721

[3] Steinman RM, Turley S, Mellman I, Inaba K (2000) The induction of tolerance by dendritic cells that have captured apoptotic cells. J Exp Med 191: 411–416. 10662786 10.1084/jem.191.3.411 PMC2195815

[4] Chung EY, Kim SJ, Ma XJ (2006) Regulation of cytokine production during phagocytosis of apoptotic cells. Cell Res 16: 154–161. 16474428 10.1038/sj.cr.7310021

[5] Mathis D, Vence L, Benoist C (2001) beta-Cell death during progression to diabetes. Nature 414: 792–798. 11742411 10.1038/414792a

[6] Knip M, Siljander H (2008) Autoimmune mechanisms in type 1 diabetes. Autoimmun Rev 7: 550–557. 18625444 10.1016/j.autrev.2008.04.008

[7] Marin-Gallen S, Clemente-Casares X, Planas R, Pujol-Autonell I, Carrascal J, et al (2010) Dendritic cells pulsed with antigen-specific apoptotic bodies prevent experimental type 1 diabetes. Clin Exp Immunol 160: 207–214. 20030670 10.1111/j.1365-2249.2009.04082.x PMC2857943

[8] O’Brien BA, Geng X, Orteu CH, Huang Y, Ghoreishi M, et al (2006) A deficiency in the in vivo clearance of apoptotic cells is a feature of the NOD mouse. J Autoimmun 26: 104–115. 16431079 10.1016/j.jaut.2005.11.006

[9] Faleo G, Fotino C, Bocca N, Molano RD, Zahr-Akrawi E, et al (2012) Prevention of autoimmune diabetes and induction of beta-cell proliferation in NOD mice by hyperbaric oxygen therapy. Diabetes 61: 1769–1778. 22566533 10.2337/db11-0516 PMC3379675

[10] Phillips B, Giannoukakis N, Trucco M (2009) Dendritic cell-based therapy in Type 1 diabetes mellitus. Expert Rev Clin Immunol 5: 325–339. 20477010 10.1586/eci.09.8

[11] Morelli AE, Larregina AT (2010) Apoptotic cell-based therapies against transplant rejection: role of recipient’s dendritic cells. Apoptosis 15: 1083–1097. 20140521 10.1007/s10495-010-0469-9 PMC2929431

[12] Peakman M, von Herrath M (2010) Antigen-specific immunotherapy for type 1 diabetes: maximizing the potential. Diabetes 59: 2087–2093. 20805382 10.2337/db10-0630 PMC2927927

[13] Hamaguchi K, Gaskins HR, Leiter EH (1991) NIT-1, a pancreatic beta-cell line established from a transgenic NOD/Lt mouse. Diabetes 40: 842–849. 1647994 10.2337/diab.40.7.842

[14] Lyons AB, Parish CR (1994) Determination of lymphocyte division by flow cytometry. J Immunol Methods 171: 131–137. 8176234 10.1016/0022-1759(94)90236-4

[15] Irizarry RA, Hobbs B, Collin F, Beazer-Barclay YD, Antonellis KJ, et al (2003) Exploration, normalization, and summaries of high density oligonucleotide array probe level data. Biostatistics 4: 249–264. 12925520 10.1093/biostatistics/4.2.249

[16] Smyth GK (2004) Linear models and empirical bayes methods for assessing differential expression in microarray experiments. Stat Appl Genet Mol Biol 3: Article3. 16646809 10.2202/1544-6115.1027

[17] Benjamini Y, Hochberg Y (1995) Controlling the false discovery rate: A practical and powerful approach to multiple testing. J R Stat Soc Ser B 57: 289–300.

[18] Livak KJ, Schmittgen TD (2001) Analysis of relative gene expression data using real-time quantitative PCR and the 2(-Delta Delta C(T)) Method. Methods 25: 402–408. 11846609 10.1006/meth.2001.1262

[19] Voll RE, Herrmann M, Roth EA, Stach C, Kalden JR, et al (1997) Immunosuppressive effects of apoptotic cells. Nature 390: 350–351. 9389474 10.1038/37022

[20] Hilkens CM, Isaacs JD, Thomson AW (2010) Development of dendritic cell-based immunotherapy for autoimmunity. Int Rev Immunol 29: 156–183. 20199240 10.3109/08830180903281193

[21] Lauber K, Blumenthal SG, Waibel M, Wesselborg S (2004) Clearance of apoptotic cells: getting rid of the corpses. Mol Cell 14: 277–287. 15125832 10.1016/s1097-2765(04)00237-0

[22] Korns D, Frasch SC, Fernandez-Boyanapalli R, Henson PM, Bratton DL (2011) Modulation of macrophage efferocytosis in inflammation. Front Immunol 2: 57. 22566847 10.3389/fimmu.2011.00057 PMC3342042

[23] Blachere NE, Darnell RB, Albert ML (2005) Apoptotic cells deliver processed antigen to dendritic cells for cross-presentation. PLoS Biol 3: e185. 15839733 10.1371/journal.pbio.0030185 PMC1084338

[24] Adler AJ, Marsh DW, Yochum GS, Guzzo JL, Nigam A, et al (1998) CD4+ T cell tolerance to parenchymal self-antigens requires presentation by bone marrow-derived antigen-presenting cells. J Exp Med 187: 1555–1564. 9584134 10.1084/jem.187.10.1555 PMC2212299

[25] Miles K, Heaney J, Sibinska Z, Salter D, Savill J, et al (2012) A tolerogenic role for Toll-like receptor 9 is revealed by B-cell interaction with DNA complexes expressed on apoptotic cells. Proc Natl Acad Sci U S A 109: 887–892. 22207622 10.1073/pnas.1109173109 PMC3271931

[26] Kushwah R, Wu J, Oliver JR, Jiang G, Zhang J, et al (2010) Uptake of apoptotic DC converts immature DC into tolerogenic DC that induce differentiation of Foxp3+ Treg. Eur J Immunol 40: 1022–1035. 20101618 10.1002/eji.200939782 PMC4603937

[27] Lo D, Reilly CR, Scott B, Liblau R, McDevitt HO, et al (1993) Antigen-presenting cells in adoptively transferred and spontaneous autoimmune diabetes. Eur J Immunol 23: 1693–1698. 7686860 10.1002/eji.1830230744

[28] Lee M, Kim AY, Kang Y (2000) Defects in the differentiation and function of bone marrow-derived dendritic cells in non-obese diabetic mice. J Korean Med Sci 15: 217–223. 10803701 10.3346/jkms.2000.15.2.217 PMC3054605

[29] Sandahl M, Hunter DM, Strunk KE, Earp HS, Cook RS (2010) Epithelial cell-directed efferocytosis in the post-partum mammary gland is necessary for tissue homeostasis and future lactation. BMC Dev Biol 10: 122. 21192804 10.1186/1471-213X-10-122 PMC3022573

[30] Creusot RJ, Yaghoubi SS, Chang P, Chia J, Contag CH, et al (2009) Lymphoid-tissue-specific homing of bone-marrow-derived dendritic cells. Blood 113: 6638–6647. 19363220 10.1182/blood-2009-02-204321 PMC2710920

[31] Formby B, Miller N (1990) Autologous CD4 T-cell responses to ectopic class II major histocompatibility complex antigen-expressing single-cell islet cells: an in vitro insight into the pathogenesis of lymphocytic insulitis in nonobese diabetic mice. Proc Natl Acad Sci U S A 87: 2438–2442. 2138777 10.1073/pnas.87.7.2438 PMC53704

[32] Strid J, Lopes L, Marcinkiewicz J, Petrovska L, Nowak B, et al (2001) A defect in bone marrow derived dendritic cell maturation in the nonobesediabetic mouse. Clin Exp Immunol 123: 375–381. 11298122 10.1046/j.1365-2249.2001.01473.x PMC1906008

[33] Emamaullee JA, Davis J, Merani S, Toso C, Elliott JF, et al (2009) Inhibition of Th17 cells regulates autoimmune diabetes in NOD mice. Diabetes 58: 1302–1311. 19289457 10.2337/db08-1113 PMC2682686

[34] Keino H, Takeuchi M, Kezuka T, Hattori T, Usui M, et al (2006) Induction of eye-derived tolerance does not depend on naturally occurring CD4+CD25+ T regulatory cells. Invest Ophthalmol Vis Sci 47: 1047–1055. 16505040 10.1167/iovs.05-0110

[35] Hanig J, Lutz MB (2008) Suppression of mature dendritic cell function by regulatory T cells in vivo is abrogated by CD40 licensing. J Immunol 180: 1405–1413. 18209035 10.4049/jimmunol.180.3.1405

[36] Goodwin JS, Ceuppens J (1983) Regulation of the immune response by prostaglandins. J Clin Immunol 3: 295–315. 6140268 10.1007/BF00915791

[37] Ruggeri P, Nicocia G, Venza I, Venza M, Valenti A, et al (2000) Polyamine metabolism in prostaglandin E2-treated human T lymphocytes. Immunopharmacol Immunotoxicol 22: 117–129. 10737261 10.3109/08923970009016410

[38] Baratelli F, Lin Y, Zhu L, Yang SC, Heuze-Vourc’h N, et al (2005) Prostaglandin E2 induces FOXP3 gene expression and T regulatory cell function in human CD4+ T cells. J Immunol 175: 1483–1490. 16034085 10.4049/jimmunol.175.3.1483

[39] Hancock WW, Polanski M, Zhang J, Blogg N, Weiner HL (1995) Suppression of insulitis in non-obese diabetic (NOD) mice by oral insulin administration is associated with selective expression of interleukin-4 and -10, transforming growth factor-beta, and prostaglandin-E. Am J Pathol 147: 1193–1199. 7485382 PMC1869521

[40] Fadok VA, Bratton DL, Konowal A, Freed PW, Westcott JY, et al (1998) Macrophages that have ingested apoptotic cells in vitro inhibit proinflammatory cytokine production through autocrine/paracrine mechanisms involving TGF-beta, PGE2, and PAF. J Clin Invest 101: 890–898. 9466984 10.1172/JCI1112 PMC508637

[41] Jozefowski S, Biedron R, Bobek M, Marcinkiewicz J (2005) Leukotrienes modulate cytokine release from dendritic cells. Immunology 116: 418–428. 16313356 10.1111/j.1365-2567.2005.02241.x PMC1802435

[42] Nencioni A, Wesselborg S, Brossart P (2003) Role of peroxisome proliferator-activated receptor gamma and its ligands in the control of immune responses. Crit Rev Immunol 23: 1–13. 12906257 10.1615/critrevimmunol.v23.i12.10

[43] Roszer T, Menendez-Gutierrez MP, Lefterova MI, Alameda D, Nunez V, et al (2011) Autoimmune kidney disease and impaired engulfment of apoptotic cells in mice with macrophage peroxisome proliferator-activated receptor gamma or retinoid X receptor alpha deficiency. J Immunol 186: 621–631. 21135166 10.4049/jimmunol.1002230 PMC4038038

[44] Torres-Aguilar H, Aguilar-Ruiz SR, Gonzalez-Perez G, Munguia R, Bajana S, et al (2010) Tolerogenic dendritic cells generated with different immunosuppressive cytokines induce antigen-specific anergy and regulatory properties in memory CD4+ T cells. J Immunol 184: 1765–1775. 20083662 10.4049/jimmunol.0902133

[45] Suciu-Foca Cortesini N, Piazza F, Ho E, Ciubotariu R, LeMaoult J, et al (2001) Distinct mRNA microarray profiles of tolerogenic dendritic cells. Hum Immunol 62: 1065–1072. 11600212 10.1016/s0198-8859(01)00310-x

[46] Morel PA, Srinivas M, Turner MS, Fuschiotti P, Munshi R, et al (2011) Gene expression analysis of dendritic cells that prevent diabetes in NOD mice: analysis of chemokines and costimulatory molecules. J Leukoc Biol 90: 539–550. 21628331 10.1189/jlb.0311126 PMC3157898

[47] Torres-Aguilar H, Blank M, Jara LJ, Shoenfeld Y (2010) Tolerogenic dendritic cells in autoimmune diseases: crucial players in induction and prevention of autoimmunity. Autoimmun Rev 10: 8–17. 20678591 10.1016/j.autrev.2010.07.015

[48] Van Elssen CH, Vanderlocht J, Oth T, Senden-Gijsbers BL, Germeraad WT, et al (2011) Inflammation-restraining effects of prostaglandin E2 on natural killer-dendritic cell (NK-DC) interaction are imprinted during DC maturation. Blood 118: 2473–2482. 21715307 10.1182/blood-2010-09-307835

[49] Muthuswamy R, Mueller-Berghaus J, Haberkorn U, Reinhart TA, Schadendorf D, et al (2010) PGE(2) transiently enhances DC expression of CCR7 but inhibits the ability of DCs to produce CCL19 and attract naive T cells. Blood 116: 1454–1459. 20498301 10.1182/blood-2009-12-258038 PMC2938836

[50] Albert ML, Sauter B, Bhardwaj N (1998) Dendritic cells acquire antigen from apoptotic cells and induce class I-restricted CTLs. Nature 392: 86–89. 9510252 10.1038/32183

[51] Brandmaier AG, Leitner WW, Ha SP, Sidney J, Restifo NP, et al (2009) High-avidity autoreactive CD4+ T cells induce host CTL, overcome T(regs) and mediate tumor destruction. J Immunother 32: 677–688. 19561540 10.1097/CJI.0b013e3181ab1824 PMC2747815

