# Supplementary material for: Efferocytosis Promotes Suppressive Effects on Dendritic Cells through Prostaglandin E2 Production in the Context of Autoimmunity
Source: PLoS One. 2013 May 15;8(5):e63296. doi: 10.1371/journal.pone.0063296 (PMC3654963; doi:10.1371/journal.pone.0063296)
Supplement: Table S1 — Differentially expressed genes in DCs after efferocytosis of islet cells. (DOCX) [file pone.0063296.s002.docx]

**Table S1.** Differentially expressed genes in DCs after efferocytosis of islet cells.

| **Category** | **Symbol** | **Gene ID** | **Name** | **Log2 FC** |
| --- | --- | --- | --- | --- |
| ***Actin-Based Motility*** | **Ankrd44** | 329154 | ankyrin repeat domain 44 | -0.52 |
|  | **Enah** | 13800 | enabled homolog | -0.53 |
|  | **Fmnl1** | 57778 | formin-like 1 | -0.50 |
|  | **Fscn1** | 14086 | fascin homolog 1, actin bundling protein | -0.65 |
|  | **Myo1b** | 17912 | myosin IB | 0.68 |
|  | **Ssh2** | 237860 | slingshot homolog 2 | -0.85 |
|  | **Tns3** | 319939 | Tensin 3 | 0.59 |
| ***Adhesion*** | **Adora3** | 11542 | adenosine A3 receptor | -0.72 |
|  | **Amica1** | 270152 | adhesion molecule, interacts with CXADR antigen 1 | -0.88 |
|  | **Ccdc80** | 67896 | coiled-coil domain containing 80 | -0.88 |
|  | **Cd34** | 12490 | CD34 antigen | -1.26 |
|  | **Cd69** | 12515 | CD69 antigen | -0.68 |
|  | **Cldn1** | 12737 | claudin 1 | -1.05 |
|  | **Flrt2** | 399558 | fibronectin leucine rich transmembrane protein 2 | 0.50 |
|  | **Flrt3** | 71436 | fibronectin leucine rich transmembrane protein 3 | 0.50 |
|  | **Hepacam2** | 101202 | HEPACAM family member 2 | -0.50 |
|  | **Pcdh7** | 54216 | protocadherin 7 | 0.66 |
|  | **Pdpn** | 14726 | podoplanin | 0.48 |
| ***Ag Presentation & Coestimulation*** | **Adra2a** | 11551 | adrenergic receptor, alpha 2a | -0.55 |
|  | **Cd74** | 16149 | invariant polypeptide of MHC complex, class II antigen-associated | -0.93 |
|  | **Cd80** | 12519 | CD80 antigen | -0.54 |
|  | **Cd83** | 12522 | CD83 antigen | -1.59 |
|  | **Cd86** | 12524 | CD86 antigen | -0.46 |
|  | **Clec2i** | 93675 | C-type lectin domain family 2, member i | -0.89 |
|  | **Fgl2** | 14190 | fibrinogen-like protein 2 | -0.76 |
|  | **H2-Ab1** | 14961 | histocompatibility 2, class II antigen A, beta 1 | -1.28 |
|  | **H2-DMb2** | 15000 | histocompatibility 2, class II, locus Mb2 | -0.63 |
|  | **H2-Eb1** | 14969 | histocompatibility 2, class II antigen E beta | -1.23 |
|  | **H2-M2** | 14990 | histocompatibility 2, M region locus | 0.50 |
|  | **Il4i1** | 14204 | interleukin 4 induced 1 | -1.42 |
|  | **Klrb1f** | 232408 | killer cell lectin-like receptor subfamily B member 1F | -1.03 |
|  | **Pdcd1lg2** | 58205 | programmed cell death 1 ligand 2 | -0.99 |
|  | **Rab27a** | 11891 | RAB27A, member RAS oncogene family | -0.52 |
|  | **Tmem123** | 71929 | transmembrane protein 123 | -0.49 |
| ***Apoptosis*** | **Bmf** | 171543 | BCL2 modifying factor | -0.48 |
|  | **Clec4e** | 56619 | C-type lectin domain family 4, member e | 0.50 |
|  | **Clu** | 12759 | clusterin | -0.94 |
|  | **Gas5** | 14455 | growth arrest specific 5 | -0.46 |
|  | **Gpr65** | 14744 | G-protein coupled receptor 65 | -1.07 |
|  | **Rassf2** | 215653 | Ras association (RalGDS/AF-6) domain family member 2 | -0.65 |
|  | **Spint2** | 20733 | serine protease inhibitor, Kunitz type 2 | -0.49 |
|  | **Trp53i11** | 277414 | transformation related protein 53 inducible protein 11 | -0.53 |
|  | **Tspan13** | 66109 | tetraspanin 13 | -1.11 |
| ***Cell Cycle*** | **Ccnd2** | 12444 | cyclin D2 | -0.55 |
|  | **Ccng2** | 12452 | cyclin G2 | -0.51 |
|  | **Cdc14a** | 229776 | CDC14 cell division cycle 14 homolog A | -0.46 |
|  | **Ctdsp2** | 52468 | CTD small phosphatase 2 | -0.48 |
|  | **Dna2** | 327762 | DNA replication helicase 2 homolog | -0.64 |
|  | **Hemk1** | 69536 | HemK methyltransferase family member 1 | -0.64 |
|  | **Ms4a6d** | 68774 | membrane-spanning 4-domains, subfamily A, member 6D | 0.54 |
|  | **Plk2** | 20620 | polo-like kinase 2 | 0.54 |
|  | **Pten** | 19211 | phosphatase and tensin homolog | -0.51 |
|  | **Trp53inp1** | 60599 | transformation related protein 53 inducible nuclear protein 1 | -0.50 |
| ***Chemokines*** | **Ccl12** | 20293 | chemokine (C-C motif) ligand 12 | 0.62 |
|  | **Ccl17** | 20295 | chemokine (C-C motif) ligand 17 | -1.20 |
|  | **Ccl2** | 20296 | chemokine (C-C motif) ligand 2 | 0.83 |
|  | **Ccl22** | 20299 | chemokine (C-C motif) ligand 22 | -2.18 |
|  | **Ccl3** | 20302 | chemokine (C-C motif) ligand 3 | 0.48 |
|  | **Ccl4** | 20303 | chemokine (C-C motif) ligand 4 | 0.52 |
|  | **Ccl5** | 20304 | chemokine (C-C motif) ligand 5 | -2.24 |
|  | **Ccl7** | 20306 | chemokine (C-C motif) ligand 7 | 1.04 |
|  | **Ccr2** | 12772 | chemokine (C-C motif) receptor 2 | -1.03 |
|  | **Ccr7** | 12775 | chemokine (C-C motif) receptor 7 | -2.42 |
|  | **Cx3cl1** | 20312 | chemokine (C-X3-C motif) ligand 1 | -0.72 |
|  | **Cxcl1** | 14825 | chemokine (C-X-C motif) ligand 1 | 0.74 |
|  | **Cxcl5** | 20311 | chemokine (C-X-C motif) ligand 5 | 1.57 |
|  | **Cxcr2** | 12765 | chemokine (C-X-C motif) receptor 2 | -0.55 |
|  | **Ppbp** | 57349 | pro-platelet basic protein | 0.54 |
| ***Cytokines*** | **Il18rap** | 16174 | interleukin 18 receptor accessory protein | -0.80 |
|  | **Il1a** | 16175 | interleukin 1, alpha | 0.84 |
|  | **Il1r2** | 16178 | interleukin 1 receptor, type II | -1.75 |
|  | **Il2ra** | 16184 | interleukin 2 receptor, alpha chain | -1.37 |
|  | **Tnf** | 21926 | tumor necrosis factor | 0.78 |
|  | **Tnfrsf18** | 21936 | tumor necrosis factor receptor superfamily, member 18 | -0.50 |
|  | **Tnfrsf9** | 21942 | tumor necrosis factor receptor superfamily, member 9 | -1.52 |
|  | **Tnfsf4** | 22164 | tumor necrosis factor (ligand) superfamily, member 4 | -1.18 |
|  | **Tnfsf8** | 21949 | tumor necrosis factor (ligand) superfamily, member 8 | -0.64 |
| ***Immunoregulation*** | **Arrdc3** | 105171 | arrestin domain containing 3 | -0.55 |
|  | **Asb2** | 65256 | ankyrin repeat and SOCS box-containing 2 | -0.72 |
|  | **Cytip** | 227929 | cytohesin 1 interacting protein | -1.33 |
|  | **Dok7** | 231134 | docking protein 7 | 0.54 |
|  | **Fst** | 14313 | follistatin | 0.59 |
|  | **Htra1** | 56213 | HtrA serine peptidase 1 | -0.89 |
|  | **Klk1b11** | 16613 | kallikrein 1-related peptidase b11 | -1.00 |
|  | **Klk1b21** | 16616 | kallikrein 1-related peptidase b21 | -0.52 |
|  | **Klk1b9** | 13648 | kallikrein 1-related peptidase b9 | -0.53 |
|  | **Lilra6** | 18726 | leukocyte immunoglobulin-like receptor, subfamily A, member 6 | -0.48 |
|  | **Ly9** | 17085 | lymphocyte antigen 9 | 0.56 |
|  | **Mmp25** | 240047 | matrix metallopeptidase 25 | -1.75 |
|  | **Pkib** | 18768 | protein kinase inhibitor beta, cAMP dependent, testis specific | -0.51 |
|  | **Rcan1** | 54720 | regulator of calcineurin 1 | 0.58 |
|  | **Retnla** | 57262 | resistin like alpha | -2.09 |
|  | **Rgs18** | 64214 | regulator of G-protein signaling 18 | -0.84 |
|  | **S100a8** | 20201 | S100 calcium binding protein A8 | -0.61 |
|  | **S100a9** | 20202 | S100 calcium binding protein A9 | -0.51 |
|  | **Serpinb10** | 241197 | serpin peptidase inhibitor, clade B (ovalbumin), member 10 | -0.50 |
|  | **Serpinb2** | 18788 | serine (or cysteine) peptidase inhibitor, clade B, member 2 | 1.06 |
|  | **Serpinb6b** | 20708 | serine (or cysteine) peptidase inhibitor, clade B, member 6b | -0.55 |
|  | **Serpinb8** | 20725 | serpin peptidase inhibitor, clade B (ovalbumin), member 8 | 0.79 |
|  | **Sesn2** | 230784 | sestrin 2 | -0.58 |
|  | **Sh2d1b1** | 26904 | SH2 domain protein 1B1 | -0.60 |
|  | **Slamf6** | 30925 | SLAM family member 6 | 0.55 |
|  | **Slamf7** | 75345 | SLAM family member 7 | -0.78 |
|  | **Slfn1** | 20555 | schlafen 1 | -1.39 |
|  | **Socs2** | 216233 | suppressor of cytokine signaling 2 | -0.56 |
|  | **Stfa1** | 20861 | stefin A1 | -0.56 |
|  | **Stfa3** | 20863 | stefin A3 | -0.48 |
|  | **Tmem176a** | 66058 | transmembrane protein 176A | -0.61 |
|  | **Tmem176b** | 65963 | transmembrane protein 176B | -1.32 |
|  | **Trmt61a** | 328162 | tRNA methyltransferase 61 homolog A | 0.52 |
|  | **Vasn** | 246154 | vasorin | 0.50 |
| ***Metabolism*** | **Acat3** | 224530 | acetyl-Coenzyme A acetyltransferase 3 | -0.66 |
|  | **Acsl1** | 14081 | acyl-CoA synthetase long-chain family member 1 | 0.78 |
|  | **Akr1c18** | 105349 | aldo-keto reductase family 1, member C18 | -1.03 |
|  | **Aldh1a2** | 19378 | aldehyde dehydrogenase family 1, subfamily A2 | -1.27 |
|  | **Alox15** | 11687 | arachidonate 15-lipoxygenase | -0.62 |
|  | **B3galt2** | 26878 | UDP-Gal:betaGlcNAc beta 1,3-galactosyltransferase, polypeptide 2 | -0.78 |
|  | **Car2** | 12349 | carbonic anhydrase 2 | -0.63 |
|  | **Car4** | 12351 | carbonic anhydrase 4 | -0.55 |
|  | **Ch25h** | 12642 | cholesterol 25-hydroxylase | 1.20 |
|  | **Cyp11a1** | 13070 | cytochrome P450, family 11, subfamily a, polypeptide 1 | -0.48 |
|  | **Cyp4f18** | 72054 | cytochrome P450, family 4, subfamily f, polypeptide 18 | -0.66 |
|  | **Dgka** | 13139 | diacylglycerol kinase, alpha | -0.64 |
|  | **Dio2** | 13371 | deiodinase, iodothyronine, type II | 0.70 |
|  | **Ereg** | 13874 | epiregulin | 0.95 |
|  | **F13a1** | 74145 | coagulation factor XIII, A1 subunit | 0.65 |
|  | **F2rl2** | 14064 | coagulation factor II (thrombin) receptor-like 2 | -0.63 |
|  | **F3** | 14066 | coagulation factor III (thromboplastin, tissue factor) | 0.55 |
|  | **Fabp4** | 11770 | fatty acid binding protein 4, adipocyte | 0.76 |
|  | **Fbp1** | 14121 | fructose bisphosphatase 1 | -0.81 |
|  | **Igf1** | 16000 | insulin-like growth factor 1 (somatomedin C) | 0.83 |
|  | **Kmo** | 98256 | kynurenine 3-monooxygenase (kynurenine 3-hydroxylase) | -0.84 |
|  | **Lipe** | 16890 | lipase, hormone sensitive | -0.79 |
|  | **Lipn** | 70166 | lipase, family member N | 0.75 |
|  | **Ltc4s** | 17001 | leukotriene C4 synthase | -0.48 |
|  | **Maoa** | 17161 | monoamine oxidase A | 0.58 |
|  | **Mtmr4** | 170749 | myotubularin related protein 4 | -0.85 |
|  | **Nedd4** | 17999 | neural precursor cell expressed, developmentally down-regulated 4 | -0.72 |
|  | **P2ry10** | 78826 | purinergic receptor P2Y, G-protein coupled 10 | -1.37 |
|  | **Pde3b** | 18576 | phosphodiesterase 3B, cGMP-inhibited | -0.49 |
|  | **Pip4k2a** | 18718 | phosphatidylinositol-5-phosphate 4-kinase, type II, alpha | -0.51 |
|  | **Ppap2b** | 67916 | phosphatidic acid phosphatase type 2B | 0.52 |
|  | **Ptges** | 64292 | prostaglandin E synthase | 1.10 |
|  | **Ptgs2** | 19225 | prostaglandin-endoperoxide synthase 2 | 0.97 |
|  | **Ramp3** | 56089 | receptor (calcitonin) activity modifying protein 3 | -2.21 |
|  | **St3gal4** | 20443 | ST3 beta-galactoside alpha-2,3-sialyltransferase 4 | -0.48 |
|  | **Stfa2l1** | 268885 | stefin A2 like 1 | -1.64 |
|  | **Uck2** | 80914 | uridine-cytidine kinase 2 | -0.56 |
| ***Natural Immunity*** | **Abp1** | 76507 | amiloride binding protein 1 (amine oxidase, copper-containing) | -0.54 |
|  | **Aoah** | 27052 | acyloxyacyl hydrolase (neutrophil) | 0.55 |
|  | **Apobec1** | 11810 | apolipoprotein B mRNA editing enzyme, catalytic polypeptide 1 | 0.63 |
|  | **Cd180** | 17079 | CD180 antigen | 0.56 |
|  | **Cd209a** | 170786 | CD209a antigen | -0.46 |
|  | **Cd300e** | 217306 | CD300e antigen | -0.63 |
|  | **Cfb** | 14962 | complement factor B | 0.52 |
|  | **Clec10a** | 17312 | C-type lectin domain family 10, member A | -0.61 |
|  | **Colec12** | 140792 | collectin sub-family member 12 | 0.50 |
|  | **Epx** | 13861 | eosinophil peroxidase | -0.54 |
|  | **Ifitm6** | 213002 | interferon induced transmembrane protein 6 | -1.41 |
|  | **Lcn2** | 16819 | lipocalin 2 | -1.69 |
|  | **Marco** | 17167 | macrophage receptor with collagenous structure | 1.07 |
|  | **Mgl2** | 216864 | macrophage galactose N-acetyl-galactosamine specific lectin 2 | -1.23 |
|  | **Mrc1** | 17533 | mannose receptor, C type 1 | 0.54 |
|  | **Mx1** | 17857 | myxovirus (influenza virus) resistance 1 | -0.55 |
|  | **Myo10** | 17909 | myosin X | 0.50 |
|  | **Pglyrp1** | 21946 | peptidoglycan recognition protein 1 | -0.68 |
|  | **Prg2** | 19074 | proteoglycan 2, bone marrow | -1.06 |
|  | **Ptx3** | 19288 | pentraxin 3, long | 0.54 |
|  | **Saa3** | 20210 | serum amyloid A 3 | 0.49 |
|  | **Sp140** | 434484 | Sp140 nuclear body protein | -0.61 |
|  | **Trim13** | 66597 | tripartite motif-containing 13 | 0.49 |
| ***NIT-1 cells*** | **Blnk** | 17060 | B-cell linker | 0.85 |
|  | **Cck** | 12424 | Cholecystokinin | 1.03 |
|  | **Cpe** | 12876 | carboxypeptidase E | 0.56 |
|  | **Dcbld2** | 73379 | discoidin, CUB and LCCL domain containing 2 | 0.72 |
|  | **Iapp** | 15874 | islet amyloid polypeptide | 1.70 |
|  | **Igf2** | 16002 | insulin-like growth factor 2 | 0.69 |
|  | **Ins1** | 16333 | insulin I | 0.55 |
|  | **Ins2** | 16334 | insulin II | 1.82 |
|  | **Maob** | 109731 | monoamine oxidase B | 0.51 |
|  | **Mela** | 17276 | melanoma antigen | 1.99 |
|  | **Nt5dc2** | 70021 | 5'-nucleotidase domain containing 2 | 0.53 |
|  | **Plxna2** | 18845 | plexin A2 | 0.48 |
|  | **Sst** | 20604 | somatostatin | 2.17 |
|  | **Tspan7** | 21912 | tetraspanin 7 | 1.37 |
| ***Other*** | **6330407A03Rik** | 70720 | RIKEN cDNA 6330407A03 gene | -0.46 |
|  | **A530032D15Rik** | 381287 | RIKEN cDNA A530032D15Rik gene | -0.52 |
|  | **A530064D06Rik** | 328830 | RIKEN cDNA A530064D06 gene | -0.51 |
|  | **A930001N09Rik** | 77128 | RIKEN cDNA A930001N09 gene | -0.61 |
|  | **AB041803** | 232685 | cDNA sequence AB041803 | -0.50 |
|  | **AI607873** | 226691 | expressed sequence AI607873 | 0.91 |
|  | **Arap2** | 212285 | ArfGAP with RhoGAP domain, ankyrin repeat and PH domain 2 | -0.59 |
|  | **BC018473** | 193217 | cDNA sequence BC018473 | -1.08 |
|  | **D16Ertd472e** | 67102 | DNA segment, Chr 16, ERATO Doi 472, expressed | -0.50 |
|  | **Fam117b** | 72750 | family with sequence similarity 117, member | -0.57 |
|  | **Fam169b** | 434197 | family with sequence similarity 169, member B | -0.48 |
|  | **Fam198b** | 68659 | family with sequence similarity 198, member B | 0.79 |
|  | **Fam20c** | 80752 | family with sequence similarity 20, member C | 0.72 |
|  | **Fam49a** | 76820 | family with sequence similarity 49, member A | -0.54 |
|  | **Fam60a** | 56306 | family with sequence similarity 60, member A | -0.48 |
|  | **Fam60a** | 56306 | family with sequence similarity 60, member A | -0.50 |
|  | **Fam82a1** | 381110 | family with sequence similarity 82, member A1 | 0.49 |
|  | **Gapt** | 238875 | Grb2-binding adaptor, transmembrane | -0.69 |
|  | **Klrb1b** | 80782 | killer cell lectin-like receptor subfamily B member 1B | -0.93 |
|  | **Ms4a6c** | 73656 | membrane-spanning 4-domains, subfamily A, member 6C | 0.62 |
|  | **Phxr4** | 18689 | per-hexamer repeat gene 4 | -0.51 |
|  | **Pmp22** | 18858 | peripheral myelin protein 22 | 0.63 |
|  | **Rab44** | 442827 | RAB44, member RAS oncogene family | -0.49 |
|  | **Rogdi** | 66049 | rogdi homolog | -0.47 |
|  | **Tarm1** | 245126 | T cell-interacting, activating receptor on myeloid cells 1 | -0.53 |
|  | **Tm4sf19** | 277203 | transmembrane 4 L six family member 19 | 0.53 |
|  | **Tmem149** | 101883 | transmembrane protein 149 | -0.50 |
|  | **Tmtc2** | 278279 | transmembrane and tetratricopeptide repeat containing 2 | -0.71 |
|  | **Tspan33** | 232670 | tetraspanin 33 | -0.49 |
|  | **Tubb2a** | 22151 | tubulin, beta 2A | 0.57 |
| ***Signaling*** | **Angptl2** | 26360 | angiopoietin-like 2 | 0.55 |
|  | **Arhgap26** | 71302 | Rho GTPase activating protein 26 | -0.49 |
|  | **Arhgef12** | 69632 | Rho guanine nucleotide exchange factor (GEF) 12 | -0.46 |
|  | **Dusp4** | 319520 | dual specificity phosphatase 4 | 0.69 |
|  | **Eepd1** | 67484 | endonuclease/exonuclease/phosphatase family domain containing 1 | 0.52 |
|  | **Fyn** | 14360 | Fyn proto-oncogene | -0.51 |
|  | **Gpr114** | 382045 | G protein-coupled receptor 114 | -0.60 |
|  | **Gpr126** | 215798 | G protein-coupled receptor 126 | -0.65 |
|  | **Gpr171** | 229323 | G protein-coupled receptor 171 | -0.85 |
|  | **Gpr55** | 227326 | G protein-coupled receptor 55 | -0.47 |
|  | **Gpr97** | 54672 | G protein-coupled receptor 97 | -0.51 |
|  | **Gprc5c** | 70355 | G protein-coupled receptor, family C, group 5, member C | -0.52 |
|  | **Jak2** | 16452 | Janus kinase 2 | -1.04 |
|  | **Lphn2** | 99633 | latrophilin 2 | 0.59 |
|  | **Map3k14** | 53859 | mitogen-activated protein kinase kinase kinase 14 | -0.59 |
|  | **Mapk13** | 26415 | mitogen-activated protein kinase 13 | -0.49 |
|  | **Met** | 17295 | met proto-oncogene | 0.68 |
|  | **Olfr111** | 545205 | olfactory receptor 111 | 0.73 |
|  | **Olfr566** | 258168 | olfactory receptor 566 | 0.51 |
|  | **Pdgfa** | 18590 | platelet derived growth factor, alpha | 0.60 |
|  | **Pdgfb** | 18591 | platelet-derived growth factor beta polypeptide | 0.80 |
|  | **Pgf** | 18654 | Placental growth factor | 0.68 |
|  | **Pik3cg** | 30955 | phosphoinositide-3-kinase, catalytic, gamma polypeptide | -0.61 |
|  | **Rabgap1l** | 29809 | RAB GTPase activating protein 1-like | -0.73 |
|  | **Rasgef1b** | 320292 | RasGEF domain family, member 1B | 0.64 |
|  | **Rasgrp4** | 233046 | RAS guanyl releasing protein 4 | -0.58 |
|  | **Rgl1** | 19731 | ral guanine nucleotide dissociation stimulator,-like 1 | 0.55 |
|  | **Rhob** | 11852 | ras homolog gene family, member B | 0.54 |
|  | **Rhof** | 23912 | ras homolog gene family, member f | -0.48 |
|  | **Samsn1** | 67742 | SAM domain, SH3 domain and nuclear localization signals, 1 | -0.61 |
|  | **Stk39** | 53416 | serine/threonine kinase 39, STE20/SPS1 homolog | -0.67 |
|  | **Vrk1** | 22367 | vaccinia related kinase 1 | -0.47 |
| ***Solute Transporter*** | **Aqp9** | 64008 | aquaporin 9 | -0.79 |
|  | **Atp2a3** | 53313 | ATPase, Ca++ transporting, ubiquitous | -0.54 |
|  | **Cacnb3** | 12297 | calcium channel, voltage-dependent, beta 3 subunit | -1.30 |
|  | **Lpcat1** | 210992 | lysophosphatidylcholine acyltransferase 1 | 0.50 |
|  | **Mcoln2** | 68279 | mucolipin 2 | 0.57 |
|  | **Ms4a7** | 109225 | membrane-spanning 4-domains, subfamily A, member 7 | 0.57 |
|  | **Nup210** | 54563 | nucleoporin 210 | -0.46 |
|  | **Slc16a7** | 20503 | solute carrier family 16 (monocarboxylic acid transporters), member 7 | 0.50 |
|  | **Slc24a3** | 94249 | solute carrier family 24 (sodium/potassium/calcium exchanger), member 3 | -0.54 |
|  | **Slc36a2** | 246049 | solute carrier family 36 (proton/amino acid symporter), member 2 | 0.58 |
|  | **Slc40a1** | 53945 | solute carrier family 40 (iron-regulated transporter), member 1 | 0.73 |
|  | **Slc7a5** | 20539 | solute carrier family 7 (cationic amino acid transporter, y+ system), member 5 | -0.69 |
|  | **Slc7a8** | 50934 | solute carrier family 7 (amino acid transporter, L-type), member 8 | 0.56 |
|  | **Slco3a1** | 108116 | solute carrier organic anion transporter family, member 3a1 | -0.48 |
|  | **Slco4a1** | 108115 | solute carrier organic anion transporter family, member 4a1 | 0.64 |
|  | **Snx16** | 74718 | sorting nexin 16 | 0.48 |
|  | **Tacstd2** | 56753 | tumor-associated calcium signal transducer 2 | -0.67 |
| ***Transcription Factor*** | **Bhlhe41** | 79362 | basic helix-loop-helix family, member e41 | 0.77 |
|  | **Btg2** | 12227 | B-cell translocation gene 2, anti-proliferative | -0.71 |
|  | **Ifi204** | 15951 | interferon activated gene 204 | 0.51 |
|  | **Irf4** | 16364 | interferon regulatory factor 4 | -0.80 |
|  | **Irg1** | 16365 | immunoresponsive gene 1 | 0.76 |
|  | **Nfe2** | 18022 | nuclear factor, erythroid derived 2 | -0.51 |
|  | **Nfil3** | 18030 | nuclear factor, interleukin 3, regulated | -0.82 |
|  | **Nr4a3** | 18124 | nuclear receptor subfamily 4, group A, member 3 | -1.57 |
|  | **Sp140** | 434484 | Sp140 nuclear body protein | -0.58 |
|  | **Stat4** | 20849 | signal transducer and activator of transcription 4 | -1.27 |
|  | **Trps1** | 83925 | trichorhinophalangeal syndrome I | -0.69 |
|  | **Vdr** | 22337 | vitamin D (1,25- dihydroxyvitamin D3) receptor | -0.92 |
|  | **Vgll4** | 232334 | vestigial like 4 | -0.54 |
|  | **Xbp1** | 22433 | X-box binding protein 1 | -0.63 |
|  | **Zc3h12c** | 244871 | zinc finger CCCH type containing 12C | 0.66 |
|  | **Zfp217** | 228913 | zinc finger protein 217 | -0.69 |
